# Why are Massachusetts opioid prescribing rates higher in rural versus urban areas?

**DOI:** 10.1371/journal.pone.0349247

**Published:** 2026-05-27

**Authors:** Alicia Sasser Modestino, Gary J. Young, Md Mahmudul Hasan, Jiesheng Shi, Md Noor E. Alam

**Affiliations:** 1 Department of Economics, School of Public Policy and Urban Affairs, Northeastern University, Boston, Massachusetts, United States of America; 2 Strategic Management and Healthcare Systems, D’Amore McKim School of Business, and Northeastern Center for Health Policy and Healthcare Research, Bouve College of Health Sciences at Northeastern University, Boston, Massachusetts, United States of America; 3 College of Pharmacy Department of Information Systems and Operations Management, Warrington College of Business, University of Florida, Gainesville, Florida, United States of America; 4 Department of Mechanical and Industrial Engineering, College of Engineering, Northeastern University, Boston, Massachusetts, United States of America; 5 Mechanical and Industrial Engineering, College of Engineering, Northeastern University, Boston, Massachusetts, United States of America; Virginia Polytechnic Institute and State University, UNITED STATES OF AMERICA

## Abstract

**Aims:**

To examine the relationship between geographical density and opioid prescribing in a model that simultaneously accounts for both supply and demand side factors.

**Design:**

Using the Massachusetts All-Payer Claims (MAPC) Database, we capture individual patient-level characteristics and combine this with county-level data on economic conditions and health care delivery systems to examine the relationship between geographical density and opioid prescribing.

**Population and Setting:**

Commercially insured population residing in Massachusetts between 2010 and 2014.

**Methodology:**

Using a logistic regression framework, we determine the likelihood that an individual patient with one of the three conditions (back pain, joint disease, or car accident) will receive an opioid prescription and how this varies by geographic density between patients. We also perform a two-way Blinder-Oaxaca decomposition to understand whether it is the endowments (mean levels) of these factors or the strength of the relationship (coefficients) of those factors that is more important in explaining the differences in opioid prescribing across metropolitan and non-metropolitan areas. Finally, we also explore interactions between the demand and supply side factors.

**Findings:**

We find that patients in non-metropolitan areas were 10 percentage points more likely to receive an opioid prescription. About half of this differential is attributable to the underlying health of the local population. Focusing on three prevalent conditions for which an opioid is commonly prescribed (back pain, joint disease, and car accidents), we find that a little less than half of the remaining gap can be explained by supply side factors, such as differences in the health care delivery system. On the demand side both demographics, particularly veteran status, and health insurance type were important factors. Roughly 80 percent of the difference in opioid prescribing rates can be explained by the inclusion of both sets of covariates. Allowing for the interaction of some demand-side (e.g., working in a physically demanding occupation) and supply-side (e.g., healthcare delivery system) variables further reduces this differential to be less than half of a percentage point and statistically insignificant.

**Conclusions:**

Our findings suggest economic conditions, such as the type of working conditions that patients might experience, interact with the healthcare system in unforeseen ways and that more targeted interventions can reduce the persistent gap in opioid prescribing among more and less densely populated areas, with possible downstream impacts on overdose and mortality.

## Introduction

According to the latest data available from the 2022 National Survey on Drug Use and Health, people living in rural (non-metropolitan) areas use opioids at a higher rate (31.7 percent) than those living in urban (metropolitan) areas (26.5 percent), with even higher rates (36.0 percent) among those living in completely rural areas [1]. Previous studies have found that this disparity in opioid use is linked to greater prescribing, showing that the odds that a patient in a non-metropolitan area is prescribed an opioid is up to 50 percent higher than it is for a similar patient in an urban area [2] and that living in a non-metropolitan area is also associated with higher *amounts* of prescribed opioids [3]. More importantly, higher rates of prescribing opioids in non-metropolitan areas have been shown to have important consequences for mortality, with researchers finding that lower population density is associated with higher overdose rates [4].

This disparity in opioid prescribing by geography is present even when comparing patients that have similar characteristics, receive care in similar clinical settings, have the same type of insurance coverage and are subject to the same state regulations. For example, among veterans receiving care through the Veterans Health Administration, per capita opioid utilization was 30 percent higher among non-metropolitan versus metropolitan patients [5]. Among Medicare Part D recipients, opioid prescribing rates ranged from 3 percent in more densely populated states such as New York to 11 percent in less densely populated states such as Utah [6]. In addition, this geographic variation in the prevalence of prescribed opioids across counties is greater than the geographic variation observed for other Medicare services related to pain conditions such as prescribing of other types of drugs and performing surgical procedures to reduce pain (e.g., lumbar fusion) [7].

Moreover, the disparity in opioid prescribing across counties with varying density has even been documented within states, such as Massachusetts, suggesting that these differences are not limited to certain states or regions that are predominantly either metropolitan or non-metropolitan nor having particular regulatory environments. For example, prior research shows that Massachusetts counties with substantial rural populations in the western part of the state, such as Berkshire County, had opioid prescribing rates that were twice that of urban counties, such as Suffolk, which encompasses Boston, the state’s largest metro area [8]. A concomitant disparity in the prevalence of opioid use disorder was also found across urban and rural counties in the state [9].

This study aims to examine the relationship between geographical density and opioid prescribing in a model that simultaneously accounts for both supply and demand side factors. To date, previous studies have not been able to quantify the relative importance of each set of factors nor whether dynamic interactions exist between the demand and supply side. This has left policymakers with the unenviable task of pursuing multiple policies with little knowledge of the tradeoffs involved in the hopes of having an immediate impact on an ever-growing problem that has only been exacerbated further by the pandemic [10,11].

## Theoretical framing

We draw on a number of studies over the past decade that have examined a variety of independent factors affecting opioid prescribing rates by geographical density to inform our empirical model of both the demand and supply side of the market. On the demand side, researchers have shown that differences in patient composition, economic distress, and opioid use disorder have all played a role in explaining the disparity in opioid prescribing rates across rural (non-metropolitan) and urban (metropolitan) areas. On the supply side, differences in access to insurance, the health care delivery system, and physician prescribing patterns have also been shown to be important factors. Below we discuss the research evidence underlying the theoretical justification for incorporating each of these factors into our statistical analysis.

### Demand-side explanations

On the demand (patient) side, studies have shown that rural populations have a higher share of people among whom opioid prescribing is higher or for whom duration of treatment is greater due to underlying health conditions. For example, previous work has shown that non-metropolitan counties tend to have larger populations of older adults who have a higher prevalence of conditions associated with pain such as diabetes and arthritis [3,12]. Higher rates of opioid prescribing are also associated with areas that have a larger percentage of non-Hispanic whites, a population that is more prevalent in non-metropolitan areas [3].

Economic factors have also been linked to differences in opioid prescribing by geography. For example, non-metropolitan areas have higher employment shares in industries that are more likely to lead to injuries that require pain medication such as construction, production, and transportation [13]. In particular, workers employed in mining and construction industries are more likely than workers in other industries to receive opioids when receiving a prescription for pain medication and more likely to receive opioids on a longer-term basis and at higher doses [14]. Among non-metropolitan counties, the highest drug mortality rates are disproportionately concentrated in counties dependent on mining and service sector jobs that also have high rates of opioid prescribing and greater use of fentanyl [15,16].

In addition, the link between economic distress and mental health has also been shown to drive differences in opioid seeking behavior on the part of patients in rural versus urban areas. Non-metropolitan areas typically have lower labor force participation rates, slower employment growth, and higher unemployment rates among prime-working-age adults, which contribute to higher poverty rates in non-metropolitan (15.4 percent) versus metropolitan (11.9 percent) areas [13]. Large job losses and stagnant wages, such as those observed in non-metropolitan areas, have been linked to individuals being more likely to engage in substance use to alleviate depression related to economic hardships [17]. Moreover, adults residing in rural geographic locations receive mental health treatment less frequently and often with providers with less specialized training, when compared to those residing in metropolitan locations [18,19].

Finally, prior work has shown that the problem of economic distress, mental health, and the opioid crisis have become intertwined—with potentially deadly consequences. Nearly half of prime age men not in the labor force take pain medication on a daily basis, and in nearly two-thirds of these cases they take prescription pain medication [20]. One study found that for every $10,000 reduction in net income per capita, the rate of opioid overdose increases by 10 percent [4]. Another found that opioid deaths and emergency department visits are predicted to rise when county unemployment rates temporarily increase [21]. And these “deaths of despair”—drug overdoses, alcohol-related liver disease, and suicide—occur more frequently among adults without a college degree in non-metropolitan areas [22].

### Supply-side explanations

On the supply (provider) side, studies have shown that differences in access to health care by insurance type and disparities in the health care delivery system play a role in opioid prescribing differences by geography. For example, opioid prescribing is higher in areas with greater Medicaid enrollment [3] and within states, Medicaid generally plays a larger role in non-metropolitan areas [23]. In terms of the health care delivery system, differences in both the types of facilities as well as the treatments available have been shown to contribute to the disparity in opioid prescribing by geography. For example, fewer hospital beds in non-metropolitan areas often leads to more rapid or frequent discharge to skilled nursing facilities after surgical procedures, and these facilities have been shown to increase the likelihood of receiving an opioid prescription [24]. Moreover, there are fewer pain specialists in non-metropolitan areas so patients are more likely to see a primary care physician such as a general or family practitioner. Prior work has shown that primary care providers account for nearly half of all dispensed opioid prescriptions [25] and report multiple difficulties in weaning patients from chronic opioids, including medical contraindications of nonopioid alternatives and difficulty justifying weaning by stable long-term patients [26]. In addition, non-metropolitan residents have been shown to be less likely to use self-care interventions (yoga, meditation, exercise, acupuncture, relaxation techniques) compared with metropolitan residents, reportedly resulting in a 24 percentage-point differential in the likelihood of taking an opioid for pain relief [27].

The prescribing habits of physicians themselves have also been shown to be a factor on the supply side, becoming a key policy lever for states combatting the opioid crisis. Studies reveal wide variation in opioid prescribing in terms of which conditions, how often, and how much, resulting in only weak consensus regarding the appropriate use of opioids for treating pain [7]. Even within individual hospitals, prior research finds that rates of opioid prescribing vary widely between low-intensity and high-intensity prescribers, with long-term opioid use being 30 percent higher among patients treated by high-intensity prescribers [28]. Other studies have shown that opioid prescribing in non-metropolitan areas is strongly influenced by providers’ individual relationships with their patients [29], and that these relationships may lead to physician behavior that is less consistent with newer opioid prescribing guidelines [12].

Finally, earlier studies have confirmed that differential regulation or enforcement of physician prescribing by state is also likely to explain the disparity in opioid prescribing rates by geography. For example, the implementation of state-run prescription drug monitoring programs (PDMPs) has been shown to differ across predominantly urban versus rural states [12]. “Must access” PDMPs that require providers to use the PDMP to check a patient’s prescribing history in all circumstances, not only when they suspect abuse, are more common in high density (urban) states. Prior research demonstrates that these stricter PDMPs have been associated with stronger gatekeeping effects that prevent drug seeking across similar types of providers and patients by insurance type (e.g., Medicare Part D), whereas PDMPs without such provisions are found to have no such effect [30].

### Aims and research questions

Despite the increasing amount of research on geographic differences in opioid prescribing, the relative magnitudes of each factor’s contribution to the differences in prescribing rates by geography, and whether these demand and supply factors interact with one another, remains unclear [31]. To our knowledge, no prior study has been able to simultaneously explore the range of supply-side (provider) factors while also controlling for demand-side (patient) factors due to data limitations. Our primary research questions are: (1) Why are opioid prescribing rates higher in rural versus urban areas? (2) Are demand-side or supply-side factors more important in explaining geographic differences in opioid prescribing? (3) Does the interaction between specific supply and demand factors contribute to the disparity in opioid prescribing rates across more versus less densely populated areas?

These questions are important for state public health agencies to deploy their limited resources most effectively and efficiently. If the difference in prescribing rates between urban and rural areas is largely due to supply-side factors such as health care access, delivery system characteristics, or prescribing behavior then these factors need to be prioritized to reduce excessive opioid prescribing that might contribute to dependency, abuse, overdose, or death [11]. However, if the disparity by geographical density is primarily due to demand-side characteristics such as the characteristics of the local population or the underlying economic factors in those areas then policies such as improving occupational health and safety conditions and/or expanding safety net programs to reduce the severity of periods of economic distress might be more effective. Finally, if there are dynamic interactions between specific supply- and demand-side factors, then more complementary policy approaches, such as expanding the supply of alternative pain treatments while also improving occupational health and safety measures, might enhance the efficacy of public policy interventions.

## Methods

### Data and sample selection

We capture individual patient-level characteristics using the Massachusetts All-Payer Claims (MAPC) Database which largely covers a broad-based, commercially insured population. We focus on the period of time immediately after the Great Recession (2010–2014) when there was significant variation in both opioid prescribing and economic distress across more and less densely populated areas, even within states such as Massachusetts. By examining these factors within a single state (Massachusetts), we are able to eliminate the heterogeneity associated with the differential proliferation of various state regulations that have been implemented over time, producing more precise estimates.

To further reduce the heterogeneity in patient composition across metropolitan versus non-metropolitan areas, we examine specific conditions for which patients are most likely to receive an opioid prescription. Using the full MAPC database, we first determine the top 10 most common diagnoses that an opioid is typically prescribed for, which collectively account for about half of all opioid claims in the MAPC database (See Table 1). Then, for each year in our study, we identify patients receiving a diagnosis for three of the top five of these most common conditions—back pain, joint disease, or being a passenger in a car accident. Collectively, these three diagnoses account for nearly one-quarter of opioid claims in the MAPC database. We exclude the other two diagnoses that were either less likely to occur in rural versus urban areas (e.g., Bus Occupant Injured in Transport Accident) or represented a catch-all diagnosis category (Symptoms of Ill Defined Causes).

**Table 1:** Top Diagnoses for which an Opioid is Prescribed in the MAPC Database.

First two digits of ICD-10 Code^a^	Percentage of MAPC Opioid Claims	Condition^b^	Sub-Condition
**72**	**11.59**	**Dorsopathies (Spinal Diseases) (720–724), Rheumatism (excluding spine) (725–729)**	**35% are unspecified back pains**
78	10.32	Symptoms of Ill Defined Causes (780–789) (Transient, cause cannot be identified)	28% Chest related, 24% Abdomen and Pelvis
**71**	**6.87**	**Arthropathies and Related Disorders (710–719) (Joint Diseases)**	**37% Osteoarthritis, 41% Other/unspecified**
V79	5.73	Bus occupant injured in transport accident	45% Accident with 2 or 3 wheel vehicle, 31% pedestrian
**V59**	**3.73**	**Occupant of pick-up truck or van injured**	**65% Non-collision accident like overturning**
25	2.68	Diabetes and other Endocrine Disorders (250–259)	88% Diabetes with no complication
27	2.47	Other Metabolic and Immunity Disorders (270–279)	54% Dyslipidemia
30	2.05	Neurotic Disorders (300–309) (Anxiety, Drug Dependence, Nondependent Abuse of Drugs)	34% Anxiety
42	2.11	Other forms of Heart Disease (420–429)	56% Heart arrythmia
59	2.52	Other diseases of the Urinary System (590–599)	41% Kidney Stone, 39% Other, such as UTI
			
**Total**	**50.07**		

Source: Authors’ calculations using the Massachusetts All Payer’s Claim Database.

^a^ ICD-10 codes are from the International Classification of Diseases, Tenth Edition.

^b^ Conditions that are highlighted in bold are those that were used in the analysis.

**Fig 1:**
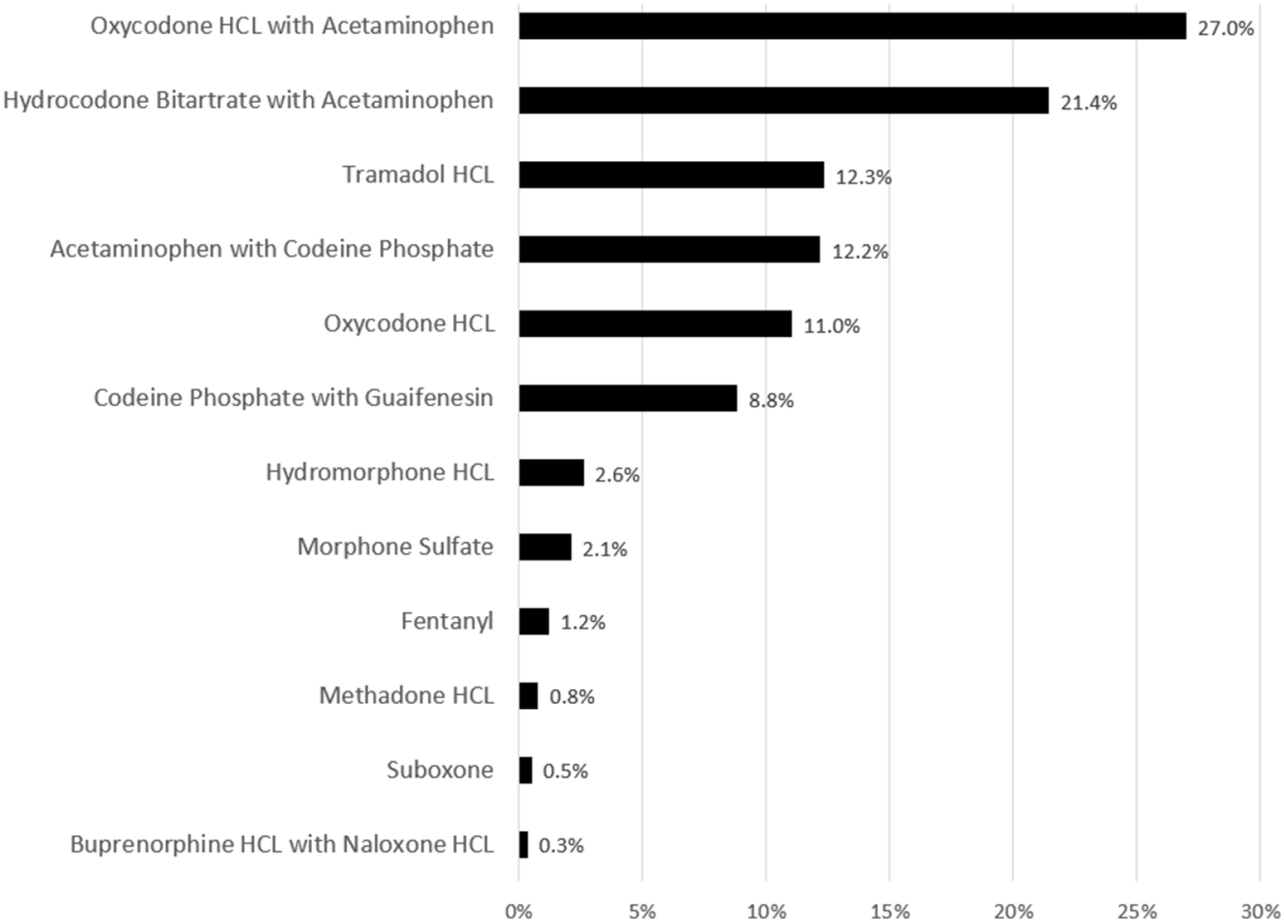
Percentage of unique opioid claims by drug prescribed, 2010. Source: Authors’ calculations using the Massachusetts All-Payers Claims Database.

We then follow patients over time in the MAPC database as they move through each stage of being diagnosed and receiving medical treatment over time, including whether or not they received an opioid as part of their treatment. First, we identify which patients received an opioid prescription based on a review of their pharmacy claims within 180 days of the first diagnosis of the three conditions that we study. Second, we restrict our analysis to the 12 most commonly prescribed opioids which account for 99 percent of all opioid claims in our database (see Fig 1). Note that we exclude individuals who were prescribed opioids that are commonly used for treating opioid use disorder, such as buprenorphine, which account for less than one percent of the sample and have no qualitative impact on our results. Third, we exclude patients who changed zip codes during our study period (2010–2014) which account for about 10 percent of unique patients in the dataset.

### Patient- and county-level variables

We then collapse the resulting claims-level dataset to the patient level and create an analytic dataset that includes both patient- and county-level characteristics. The patient-level characteristics come from the claims database and include our dependent variable which is whether or not the individual was prescribed an opioid during the 180-day treatment window as well as control variables such as basic demographics (e.g., age, sex) and insurance type (e.g., HMO/self-pay, PPO, indemnity, public, and other non-specified). We also collect the National Provider Identifier (NPI) of the clinicians involved in those claims and merge in the provider’s specialty from the NPI Registry (14 specialties).

We then merge in county-level information from two sources. First, we use the Area Health Resource File which contains information about the health care delivery system (e.g., number and type of providers and facilities per capita) to explore supply-side factors that may affect the quantity and type of treatment that are available [32]. Second, we also use the American Community Survey 5-year Data (2010–2014) in which contains population-level demographics (e.g., race, insurance coverage, and veteran status) and economic conditions (e.g., unemployment rate, poverty rate, occupational distribution) to explore county-level demand-side factors that can affect underlying health conditions and treatment preferences.

### Density measures

To explore the relationship between population density and opioid prescribing, we first determine how best to measure density within a geographic region (e.g., zip code). Although the eastern part of Massachusetts is largely metropolitan, the western part of the state and the counties that make up the Cape Cod National Seashore, including the islands of Nantucket and Martha’s Vineyard, are far less so. Focusing on this within-state geographical variation enables us to study population density on a more granular level (e.g., zip code tabulation areas) than the stark definitions of living in a metropolitan (urban) versus a non-metropolitan (rural) county.

**Fig 2:**
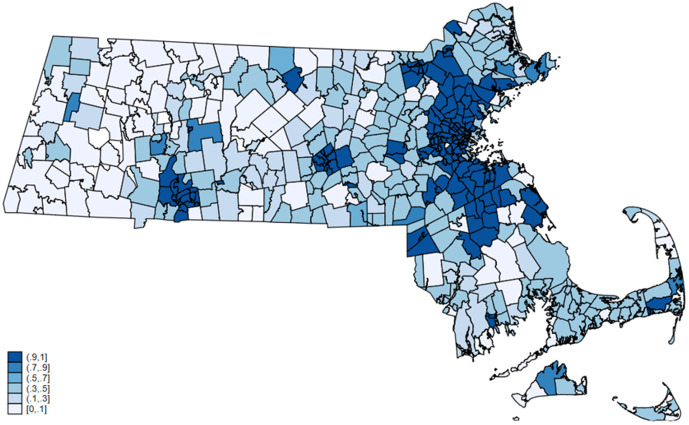
Percentage of the population in each zip code that resides in an urban ZCTA. Sources: Authors’ calculations using the Urban Area to ZIP Code Tabulation Area (ZCTA) Relationship File from the Census Bureau.

We measure density using the Urban Area to ZIP Code Tabulation Area (ZCTA) Relationship File from the Census Bureau. This file contains the population, total area, and land area for each unique urban area-ZIP Code tabulation area [33]. We then use the zip code to ZCTA crosswalk to merge these measures into our patient level dataset by zip code. We created both a dummy variable for whether the zip code contains any part of an urban (metropolitan) ZCTAs as well as a continuous variable of the percentage of the zip code population that resides in an urban ZCTA (see Fig 2). No matter which designation we use to measure density, we obtain results that were both quantitatively and qualitatively similar. For exposition purposes, we report results using the dummy variable measure and report the continuous variable results in the appendix. We also explore several other measures of county-level population density provided by the Census Bureau and the USDA Economic Research Service that yield qualitatively similar results, but do not provide as much granularity as the ZCTA designations which vary within counties.

### Statistical analysis

We then employ a logistic regression framework to determine the likelihood that an individual patient with one of the three conditions that we identified will receive an opioid prescription and how this varies by geographic density between patients. Using the Stata statistical software package version 15.1 [34], we estimate this relationship separately for each of the three conditions because they reflect a range of experiences with treating pain. For example, back pain diagnoses are more reliant on patient perceptions of pain to determine whether treatment might require an opioid. In contrast, a diagnosis for joint disease is typically confirmed with a diagnostic test (e.g., x-ray, ultrasound, MRI) that can gauge severity and provide a less subjective determination of the need for an opioid prescription. Finally, being a passenger in a car accident, a largely random event, is plausibly exogenous to underlying conditions that might require an opioid, reducing confounding influences (e.g., drug-seeking behavior) from prior diagnoses. We estimate the relationship between density and opioid prescribing using equation (1):


$$\begin{array}{cc} {\text{P}}_{\text{izct }}= & \;\alpha \;+\;\beta {\text{DENSITY}}_{\text{z}}\;+\;{\text{PATIENT}}_{\text{it}}+\;{\text{PROVIDER}}_{\text{i}}+{\text{POPULATION}}_{\text{c}}+{\text{HCDELIVERY}}_{\text{c}}\;\\ & +\;{\text{ECONOMIC}}_{\text{ct}}\;+\;{\text{YEAR}}_{\text{t}}\text{t}+\epsilon_{\text{zct i}}\; \end{array}$$ (1)

Where:

P_izct_ = indicator for whether patient *i* living in zip code *z* in county *c* in year *t* was prescribed an opioid

DENSITY_z_ = measure of density at the zip code level *z* (dummy or continuous measure);

PATIENT_i_ = vector of individual-level characteristics including age, gender, insurance type for patient *i* measured at the time of diagnosis in year t;

PROVIDER_i_ = field of specialty (e.g., primary care, surgery) of the clinician involved in the majority of the claims for the primary conditions diagnosed for patient *i;*

POPULATION_c_ = vector of county-level population characteristics for county *c* measured at the start of the period in 2010 including percent white, percent of persons less than 65 without health insurance, and percent of the population that are veterans;

HCDELIVERY_c_ = vector of health care delivery system variables for county *c* measured at the start of the period in 2010 including number of hospital beds, skilled nursing facilities, active MDs and general practitioners—all measured in per capita terms;

ECONOMIC_ct_ = vector of economic variables for county *c* measured annually from 2010–2014 including the unemployment rate for individuals aged 16 + , share of population below the poverty level, and the share of employment in physically demanding occupations including production, transportation and material moving; natural resources (e.g., mining), construction, and maintenance; and services;

YEAR_t_ = dummy variable for each year 2011–2014 (excluding the base year of 2010);

εizct = error term.

Using this model, we estimate the relationship for each condition by sequentially adding to the model each group of independent variables separately to be able to compare our results to prior studies (see S1 Fig for a conceptual model). We then include all covariates in the final specification to simultaneously determine the relative contribution of supply and demand factors that are driving the relationship between density and opioid prescribing.

Although each of these sets of factors plays a role in explaining the disparity in opioid prescribing that we observe by geography, it’s not clear whether this is simply due to these factors being more prevalent in non-metropolitan areas or whether there is a different mechanism at work [34]. For example, is it the case that greater opioid prescribing in more non-metropolitan areas is solely due to the higher share of Medicaid patients in those areas or is it also the case that Medicaid patients living in non-metropolitan areas are also more likely to receive an opioid [35,36]? To disentangle these effects, we perform a Blinder-Oaxaca decomposition which divides the gap in opioid prescribing between metropolitan and non-metropolitan areas into two components. This technique has commonly been used to study labor market discrimination [37–39]. The first component is explained by the differences in the levels (or “endowments”) of the observed related factors between metropolitan and non-metropolitan areas. The second component represents the residual part that cannot be explained by differences in the factors themselves but instead arises from the “differential effect” of the observed factors (e.g., difference in the magnitude of regression coefficients) across metropolitan and non-metropolitan areas.

We do this by first estimating separate regression models, one for metropolitan areas and one for non-metropolitan areas. We then perform the decomposition of the disparity in opioid prescribing, Y, as:


$$\begin{array}{c} \Delta \;\overset{―}{Y}=\;\left[\beta^{\text{1}}\left({\overset{―}{X}}^{\text{1}}-{\overset{―}{X}}^{\text{2}}\right)\right]\;+\;\left[{\overset{―}{X}}^{\text{2}}(\beta^{\text{1}}-\beta^{\text{2}})\right] \end{array}$$ (2)

where $\overset{―}{X}$ is a row vector of average values of the explanatory variables and β is a vector of coefficient estimates for each group 1 (metropolitan) and 2 (non-metropolitan). In this case, the coefficient estimates of group 1, β_1_, have been assumed to be as the reference.

Finally, it could be the case that the interaction between certain demand and supply side factors also contributes to the likelihood of opioid prescribing. For example, prior research shows that the majority of patients with work-related injuries are treated by primary care physicians such as general and family practitioners but most community-based physicians have little or no formal training in occupational health care [40]. Thus, opioid prescribing rates may be higher in less populated areas because patients who work in physically demanding jobs are more likely to be treated by primary care physicians who are more likely to prescribe an opioid. To test this, we also explore interactions between the demand and supply side factors such as the share of workers in physically demanding production or transportation occupations and the health care delivery system to better understand the nuances that could be useful for guiding policy solutions.

## Results

### Descriptive statistics

**Fig 3:**
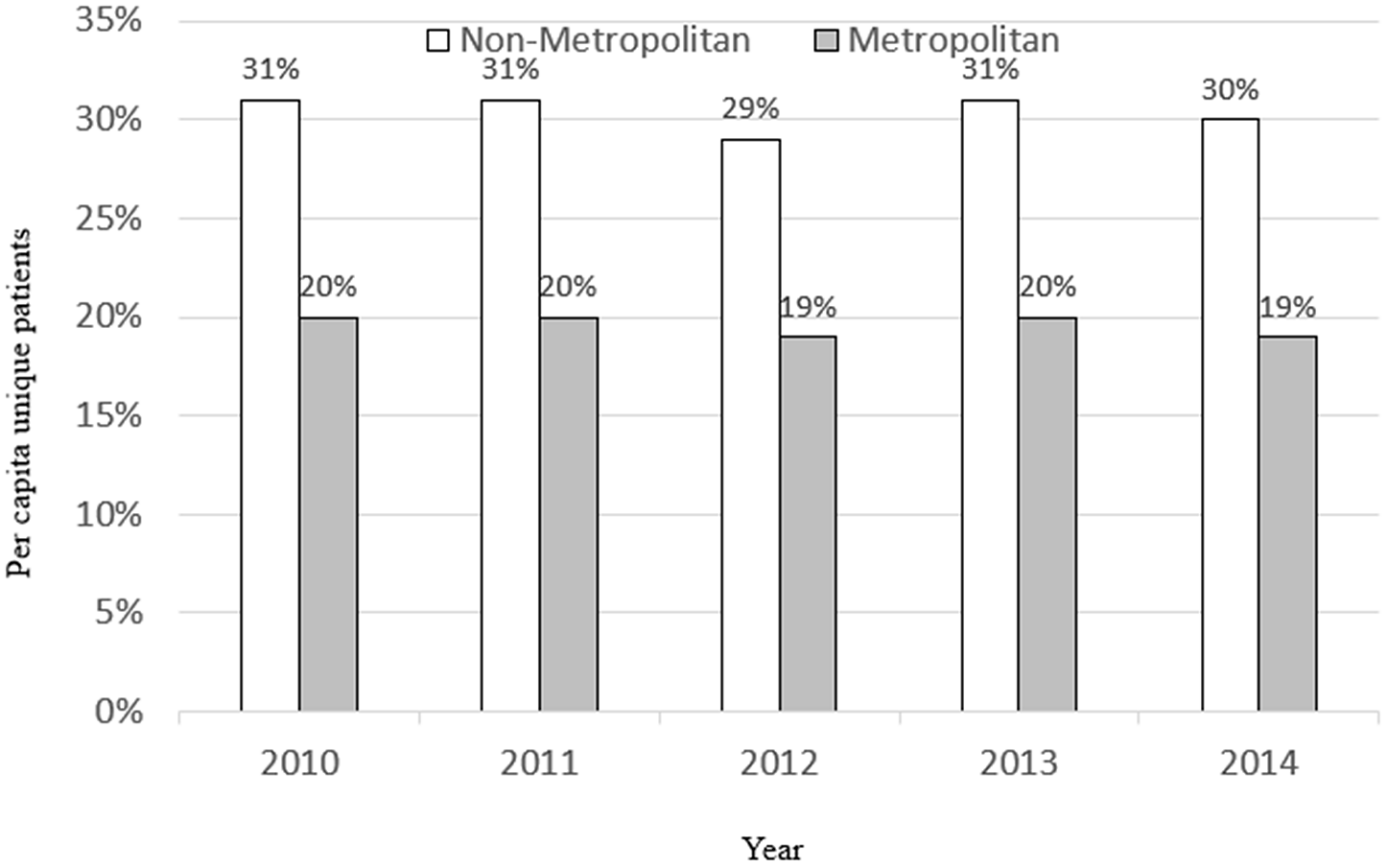
Unique patients per capita receiving an opioid prescription in Massachusetts. **all conditions**. Sources: Authors’ calculations using the Massachusetts All Payer’s Claim Database and the Urban Area to ZIP Code Tabulation Area (ZCTA) Relationship File from the Census Bureau. Note: For exposition purposes, here we are using a dummy variable (0/1) for whether the zip code contains any urban ZCTA to classify patients as living in an metropolitan versus a non-metropolitan area. In each case, the numerator is the number of unique patients who have filed a claim for an opioid prescription and the denominator is the population count in each area type (metropolitan or non-metropolitan) and each year.

**Fig 4:**
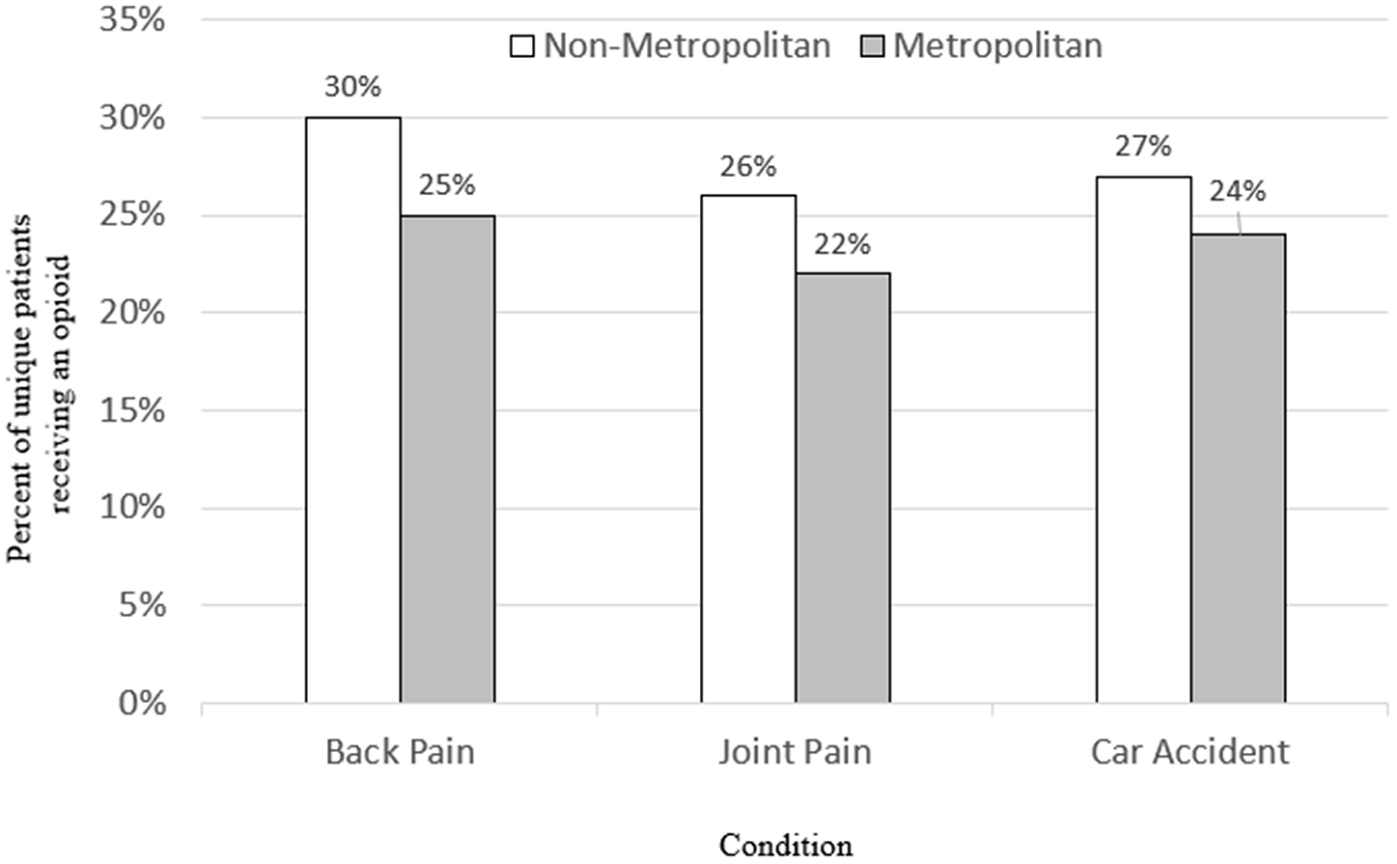
Likelihood of receiving an opioid by underlying condition in Massachusetts, 2010-2014. Sources: Authors’ calculations using the Massachusetts All Payer’s Claim Database and the Urban Area to ZIP Code Tabulation Area (ZCTA) Relationship File from the Census Bureau. Note: For exposition purposes, here we are using a dummy variable (0/1) for whether the zip code contains any urban ZCTA to classify patients as living in a metropolitan versus a non-metropolitan area. For each condition, the numerator is the number of unique patients receiving a diagnosis who have filed a claim for an opioid prescription and the denominator is the total number of patients receiving a diagnosis in each area type (metropolitan versus a non-metropolitan) between 2010 and 2014.

We find that much of the variation in opioid prescribing for urban (metropolitan) versus rural (non-metropolitan) areas is due to differences in the prevalence of the underlying clinical conditions (e.g., back pain). Using our dichotomous measure for whether the patient’s zip code contains any metropolitan ZCTA, Fig 3 displays opioid prescribing rates over time across all conditions combined, not just the top ten, for metropolitan versus non-metropolitan areas. Similar to prior studies, we find that overall prescribing rates across all conditions combined are 50 percent (10 percentage points) higher in non-metropolitan versus metropolitan areas of Massachusetts, without controlling for any covariates. However, Fig 4 shows that when we examine opioid prescribing rates across non-metropolitan and metropolitan areas for patients with similar conditions, such as back pain (ICD-10 = 72), the gap narrows to just 20 percent (5 percentage points). This indicates that roughly half of the disparity in opioid prescribing rates by geographical density in Massachusetts is driven by heterogeneity in the type of underlying clinical conditions of the population residing in those areas.

We then turn to examining which of the supply and demand side factors help explain the remaining disparity in opioid prescribing between metropolitan and non-metropolitan areas ***within*** each of our three clinical conditions. Panel A of Table 2 reports the descriptive statistics for patients diagnosed with back pain by population density for the dependent variable as well as for each of the patient-level covariates in Equation (1). Comparing the first two columns confirms that back-pain patients living in a non-metropolitan area are 4.9 percentage points more likely to be prescribed an opioid, and have a higher number of opioid claims, compared to those living in a metropolitan area. Panel A also reveals that non-metropolitan areas do indeed exhibit greater prevalence for a variety of factors that are positively correlated with higher opioid prescribing. For example, non-metropolitan areas have a higher proportion of back pain patients who have public health insurance (primarily Medicaid), and medical claims from an emergency room, primary care physician, diagnostic clinician, or end-of-life provider—characteristics linked to higher opioid prescribing. Relatedly, non-metropolitan back pain patients are less likely to have seen a pain specialist who may follow newer protocols that limit opioid prescribing or a clinician at a rehab or other medical facility who may offer alternative treatments (e.g., physical therapy) for pain.

**Table 2:** Descriptive Statistics for Sample of Patients Receiving a Diagnosis for Back Pain (2010-2014).

**Panel A. Patient Level Variables**					
	**Metropolitan (Urban)**		**Non-Metropolitan (Rural)**		**Difference in Means**
	**Mean**	**Std. Dev.**	**Mean**	**Std. Dev.**	**(Rural-Urban)**
**Density Measures**					
Lives in a non-metropolitan (rural) area	0.000	0.000	1.000	1.000	1.000***
Percent of zip code population in a metropolitan (urban) area	0.708	0.333	0.559	0.375	−0.149***
**Dependent Variables: Opioid Prescribing**					
Percent prescribed an opioid	0.247	0.431	0.295	0.456	0.049***
Number of opioid claims per patient	0.651	2.310	0.987	4.450	0.336***
**Independent Variables**					
Demographics					
Percent male	0.449	0.497	0.451	0.498	0.002
Percent age 18–24	0.082	0.275	0.079	0.270	−0.003*
Percent age 25–34	0.146	0.353	0.128	0.334	−0.018
Percent age 35–44	0.170	0.375	0.166	0.372	−0.004*
Percent age 45–54	0.216	0.411	0.219	0.413	0.003*
Percent age 55–64	0.185	0.388	0.193	0.395	0.008**
Percent age 65+	0.202	0.401	0.215	0.411	
Insurance Type					0.013***
HMO/self-pay	0.391	0.488	0.409	0.492	0.018**
PPO	0.229	0.420	0.165	0.372	−0.063***
Indemnity	0.128	0.334	0.108	0.311	−0.019**
Public (Medicare, Medicaid, VA)	0.230	0.230	0.290	0.290	0.060***
Other (not specified)	0.026	0.158	0.028	0.165	0.003
Provider Specialty					
Pain	0.017	0.130	0.010	0.100	−0.007**
Alternative pain treatment (e.g., chiropractor, physical therapy)	0.057	0.231	0.064	0.245	0.008**
Addiction	0.000	0.022	0.001	0.037	0.001
Mental health	0.015	0.122	0.016	0.125	0.001
ER/critical care	0.031	0.173	0.045	0.208	0.014***
Rehab	0.079	0.270	0.056	0.230	−0.023***
Dental	0.001	0.037	0.001	0.024	−0.001
Surgery	0.052	0.222	0.049	0.217	−0.003
General/family practitioner	0.094	0.291	0.123	0.328	0.029***
Internal medicine	0.091	0.287	0.105	0.307	0.015***
End of life (hospice, palliative care)	0.013	0.114	0.022	0.148	0.009**
Diagnostic (e.g., radiology, pathology, immunology)	0.151	0.358	0.163	0.369	0.012***
Non-MD (e.g., nurse, PA)	0.005	0.067	0.007	0.082	0.002
Medical Facility (e.g., hospital)	0.237	0.425	0.192	0.394	−0.045***
Veteran Administration / Military	0.000	0.009	0.000	−0.016	0.000
Other	0.005	0.067	0.007	0.082	0.002
Number of Patients	1,011,515		342,549		−668,966
**Panel B. County Level Variables**					
	**Metropolitan (Urban)**		**Non-Metropolitan (Rural)**		**Difference in Means**
	**Mean**	**Std. Dev.**	**Mean**	**Std. Dev.**	**(Rural-Urban)**
Population Demographics					
Percent white	0.794	0.099	0.845	0.054	0.05***
Percent persons under 65 without health insurance	0.008	0.000	0.008	0.000	0.00
Percent population veterans	0.104	0.031	0.135	0.018	0.03***
Health Care Delivery System					
Hospital beds per 10,000 residents	15.424	21.841	14.419	16.011	−1.01
Skilled nursing facility beds per 10,000 population	70.511	11.938	82.658	16.869	12.15***
Total active MDs per 10,000 population	52.490	37.374	30.973	8.882	−21.52***
General/family care specialists per 10,000 population	19.960	4.710	23.280	12.860	3.32**
Economic Conditions					
Unemployment rate, age 16+	0.067	0.014	0.079	0.014	0.01***
Poverty rate	0.106	0.042	0.132	0.029	0.03***
Percent employed in production, transportation, material moving occupations	0.083	0.021	0.120	0.020	0.04***
Percent employed in natural resource, construction, maintenance occupations	0.072	0.019	0.083	0.015	0.01**
Percent employed in service occupations	0.162	0.029	0.178	0.018	0.02**
Number of Patients	1,011,515		342,549		

Source: Patient-level variables are based on the authors’ calculations using the Massachusetts All Payer’s Claim Database and the Urban Area to ZIP Code Tabulation Area (ZCTA) Relationship File from the Census Bureau. County-level demographic and health care delivery system variables are from the Area Health Resource File. County-level labor market variables are from the American Community Survey.

Notes: For exposition purposes, here we are using a dummy variable (0/1) for whether the zip code contains any urban ZCTA to classify patients as living in a metropolitan versus a non-metropolitan area. Within this classification we also show descriptive statistics for the density variable used in our regressions which is the percentage of the ZCTA population that lives in a metropolitan area. See Table 1 for a list of covariates contained in each group. Differences are tested for significance using a two-tailed t-test of the sample between patients living in metropolitan versus non-metropolitan areas. ***Indicates statistical significance at the alpha<0.01 level, ** at the alpha<0.05 level, and * at the alpha<0.10 level.

Panel B of Table 2 confirms that patients suffering from back pain in non-metropolitan areas also live in communities with county-level characteristics that are associated with higher rates of opioid prescribing that can affect the standard of care that they receive. For example, these non-metropolitan areas have higher population shares of whites and veterans—groups that have been shown to be more likely to receive an opioid prescription, which may affect local prescribing practices. Prior research shows that this channel operates independently from the patient’s own demographic characteristics as providers often apply population-level treatment patterns across all of the patients they treat in their medical practices [11].

Similarly, patients exhibiting back pain in non-metropolitan areas also live in counties with different health care delivery systems and economic conditions. For example, these areas have fewer hospital beds but more skilled nursing facilities as well as fewer physicians per capita overall but a greater share of general practitioners—both of which have been associated with increased opioid prescrbing. In terms of economic conditions, patients suffering from back pain in non-metropolitan areas also face higher unemployment rates, greater poverty, and a labor market with a greater share of employment opportunities in physically demanding production, transportation, construction and service sector occupations—all of which have been shown to contribute to greater opioid prescribing, overdose, and mortality.

### Regression results

Continuing with our analysis of back pain patients, Table 3 estimates the relationship between density and opioid prescribing, reporting the coefficient on our dichotomous indicator of whether a patient lives in a predominantly non-metropolitan area from equation (1). We find that controlling for basic patient demographics (e.g., age, gender, and their interaction) reduces the coefficient on the population density variable by only 2.4 percent (see column (2) in Panel A of Table 3). However, controlling for patient insurance type (e.g., PPO, HMO, indemnity, self-pay, and public) reduces the coefficient on the population density variable by 23 percent, suggesting that the greater reliance of patients living in non-metropolitan areas on public health insurance is an important factor in explaining higher opioid prescribing rates. Indeed, prior research has shown that Medicaid is associated with higher rates of opioid prescribing in sparsely populated areas [35], and that this disparity largely reflects greater levels of disability and chronic illness in the populations that Medicaid serves [36]. In contrast, controlling for the specialty of the patient’s provider only slightly increases the coefficient on the density variable. Overall, the patient-level characteristics that we can measure from the medical claims data can only explain about 20 percent of the difference in urban versus rural opioid prescribing rates.

**Table 3:** Estimating the Relationship between Density and Opioid Prescribing: Back Pain (2010-2014).

Panel A. Controlling for Patient Level Variables					
	**Dependent Variable: Patient was Prescribed an Opioid (0/1)**				
	1	2	3	4	5
**Indicator for Non-Metropolitan (Rural) Area**	0.049***	0.047***	0.037***	0.052***	0.039***
	**(0.001)**	**(0.001)**	**(0.001)**	**(0.001)**	**(0.001)**
Controlling for Patient Demographics	NO	YES	NO	NO	YES
Controlling for Patient Insurance Type	NO	NO	YES	NO	YES
Controlling for Patient Provider Specialty	NO	NO	NO	YES	YES
Number of observations	1,354,064	1,354,064	1,354,064	1,354,064	1,354,064
R-squared	0.037	0.048	0.070	0.046	0.091
Percent of urban-rural difference explained	-----	−2.4%	−23.3%	6.9%	−19.2%
**Panel B. Controlling for County Level Variables**					
	**Dependent Variable: Patient was Prescribed an Opioid (0/1)**				
	1	2	3	4	5
**Indicator for Non-Metropolitan (Rural) Area**	0.049***	0.025***	0.030***	0.018***	0.011***
	**(0.001)**	**(0.001)**	**(0.001)**	**(0.001)**	**(0.001)**
Controlling for Population Demographics	NO	YES	NO	NO	YES
Controlling for Health Care Delivery System Variables	NO	NO	YES	NO	YES
Controlling for Economic Conditions	NO	NO	NO	YES	YES
Number of observations	1,354,064	1,354,064	1,354,064	1,354,064	1,354,064
R-squared	0.037	0.039	0.039	0.039	0.040
Percent of urban-rural difference explained	**-----**	−49%	−38%	−63%	−78%

Source: Patient-level variables are based on the authors’ calculations using the Massachusetts All Payer’s Claim Database and the Urban Area to ZIP Code Tabulation Area (ZCTA) Relationship File from the Census Bureau. County-level demographic and health care delivery system variables are from the Area Health Resource File. County-level labor market variables are from the American Community Survey.

Notes: See Table 1 for a list of covariates contained in each group. Each coefficient is from a separate regression.

***Indicates statistical significance at the alpha<0.01 level, ** at the alpha<0.05 level, and * at the alpha<0.10 level.

Panel B of Table 3 reveals that the county-level population covariates can explain a much larger share of the variation in opioid prescribing across metropolitan and non-metropolitan areas. Including county population demographics reduces the coefficient on the density variable by roughly half (see Column (2) of Panel B in Table 3). We also find that macro factors related to the health care delivery system and economic conditions are equally, if not more, important in explaining opioid prescribing differences across metropolitan and non-metropolitan areas. Including covariates measuring both the capacity of the health care delivery system as well as the types of facilities or providers reduces the coefficient on the population density variable by about 38 percent (see Column (3) of Panel B in Table 3). This is due in part to the greater reliance on skilled nursing facilities and general practitioners in more rural areas, both of which are associated with higher opioid prescribing

rates (See S4 Table in the appendix). Economic conditions are also an important driver, reducing the coefficient on the population density variable by about 70 percent when we account for unemployment, poverty, and the share of employment in physically demanding occupations (see Column (4) of Panel B in Table 3).

### Blinder-oaxaca decomposition

Table 4 reports the results of the two-way Blinder-Oaxaca decomposition when we include all of the individual- and county-level covariates. Roughly 80 percent of the raw difference in opioid prescribing rates is explained by the inclusion of both sets of covariates. Among the explained portion, differences in patient insurance type, county demographics and county economic conditions account for most of the disparity in opioid prescribing. When we examine the unexplained portion of the gap in prescribing rates by density, we observe that the differential effect of the healthcare delivery system and the economic conditions across rural and urban areas is significant. This is largely driven by the share of general practitioners per capita and the share of employment in physically demanding occupations as shown in S5. This suggests that greater opioid prescribing in rural versus urban areas is driven in part by different practice patterns where patients living in areas with similar healthcare settings and/or similar economic conditions are treated differently.

**Table 4:** Oaxaca Decomposition of Patient and County Factors for Back Pain Patients (2010-2014).

	Dependent Variable: Patient was Prescribed an Opioid (0/1)			
	Coefficient	Std. Error	Sig.	Percent Explained
Metropolitan (Urban)	0.247	(0.0004)		
Non-Metropolitan (Rural)	0.295	(0.0008)		
Difference	0.049	(0.0009)	***	
**Percent of difference that is:**				
Explained	0.039	(0.0010)	***	80.4%
Unexplained	0.010	(0.0013)	***	19.6%
**Percent explained due to differences in endowments:**				
Patient demographics	0.000	(0.0001)	*	0.3%
Patient insurance type	0.013	(0.0002)	***	27.1%
Patient provider specialty	−0.004	(0.0001)	***	−7.5%
County demographics	0.019	(0.0012)	***	39.9%
County health care delivery system	−0.017	(0.0017)	***	−34.2%
County economic conditions	0.027	(0.0014)	***	55.3%
Year	0.000	(0.0002)		−0.6%
**Percent unexplained due to differences in coefficients:**				
Patient demographics	−0.009	(0.0073)		−17.9%
Patient insurance type	−0.117	(0.0164)	***	−240.1%
Patient provider specialty	0.009	(0.0046)	**	18.8%
County demographics	−0.036	(0.1262)		−74.9%
County health care delivery system	−0.116	(0.0325)	***	−239.2%
County economic conditions	−0.173	(0.0644)	***	−357.2%
Year	0.010	(0.0088)		20.9%
Constant	0.442	(0.1588)	***	909.2%

Source: Patient-level variables are based on the authors’ calculations using the Massachusetts All Payer’s Claim Database and the Urban Area to ZIP Code Tabulation Area (ZCTA) Relationship File from the Census Bureau. County-level demographic and health care delivery system variables are from the Area Health Resource File. County-level labor market variables are from the American Community Survey.

Notes: See Table 1 for a list of covariates contained in each group.

***Indicates statistical significance at the alpha<0.01 level, ** at the alpha<0.05 level, and * at the alpha<0.10 level.

### Comparing across different conditions and interactions

Table 5 summarizes and compares the supply and demand side contributions across the three most common medical conditions in our study: back pain, joint pain, and car accidents. Across all three medical conditions, the results are quite similar with the demand side factors explaining a higher share of the overall difference in prescribing rates by population density. Overall, our model explains less of the variation in opioid prescribing for the set of patients who had received an opioid as a result of being a passenger in a car accident, particularly among the demand-side factors.In contrast, these demand side factors help explain both the higher prevalence as well as the greater likelihood of being prescribed an opioid for back pain and joint diseases in urban versus rural areas.

**Table 5:** Exploring Supply versus Demand Factors across Conditions (2010-2014).

	Dependent Variable: Patient was Prescribed an Opioid (0/1)		
	Back Pain	Joint Disease	Car Accident
**Indicator for Rural Area**	0.049***	0.045***	0.041***
	**(0.001)**	**(0.001)**	**(0.002)**
Covariates			
Supply Side Factors	0.028***	0.027***	0.027***
	(0.001)	(0.001)	(0.002)
Demand Side Factors	0.017***	0.016***	0.021***
	(0.001)	(0.001)	(0.002)
Both Supply and Demand Side Factors	0.009***	0.008***	0.011***
	(0.001)	(0.001)	(0.002)
Including Supply and Demand Side Interactions	0.002	0.003*	0.001
	(0.001)	(0.002)	(0.002)
Percent of urban-rural difference explained			
Supply Side Factors	−43.2%	−41.1%	−34.2%
Demand Side Factors	−65.1%	−63.7%	−48.1%
Both Supply and Demand Side	−81.9%	−82.3%	−71.9%
Including Supply and Demand Side Interactions	−96.0%	−93.8%	−97.2%
Number of observations	1,354,064	1,064,536	446,798
R-squared	0.094	0.097	0.100

Source: Patient-level variables are based on the authors’ calculations using the Massachusetts All Payer’s Claim Database and the Urban Area to ZIP Code Tabulation Area (ZCTA) Relationship File from the Census Bureau. County-level demographic and health care delivery system variables are from the Area Health Resource File. County-level labor market variables are from the American Community Survey.

Notes: Supply side factors include patient insurance type, patient provider specialty, and county-level health care delivery system.

Demand side factors include patient demographics, county-level demographics, and county-level economic conditions.

Each coefficient is from a separate regression. ***Indicates statistical significance at the alpha<0.01 level, ** at the alpha<0.05 level, and * at the alpha<0.10 level.

Despite having controlled separately for a range of demand and supply side factors, there remains a small but statistically significant difference in opioid prescribing between more versus less populated areas. Table 5 interacts the county-level health care delivery system variables with the occupation variables. When we add these interaction terms, the difference in opioid prescribing rates between more and less densely populated areas becomes statistically insignificant. This finding is also consistent with the Blinder-Oaxaca decomposition which indicated that the remaining unexplained portion of the gap was due to differential effects related to the healthcare delivery system and economic conditions.

## Discussion

### Key findings

We find that on average, patients in non-metropolitan areas in Massachusetts are 10 percentage points more likely to receive an opioid prescription. About half of this differential is due to the underlying health of the local population. When we limit our analysis to three prevalent conditions for which an opioid is commonly prescribed (back pain, joint pain, and car accidents), we find that a little less than half of the remaining gap can be explained by supply side factors, such as differences in the health care delivery system. Although demographics play a large role on the demand side, particularly veteran status, health insurance type is also an important factor. Our two-way Blinder-Oaxaca decomposition reveals that roughly 80 percent of the raw difference in opioid prescribing rates is explained by the inclusion of both sets of covariates, reducing the gap to roughly 1 percentage point.

We perform several robustness checks. First, we find similar results when using continuous measures of both the independent variable (e.g., the percent of the zip code population living in a metropolitan area) as well as the dependent variable (e.g., the number of opioid claims per patient) as show in S1 Table. Second, we also find similar results when analyzing the other two conditions: see S2 Table for joint condition estimates and S3 Table for car accident estimates.

### Mechanisms

As previously noted, there may be important interactions between demand and supply side factors that, in combination, may have greater explanatory power than either of these individual factors alone. Allowing for the interaction of some demand-side (e.g., working in a physically demanding occupation) and supply-side (e.g., healthcare delivery system) variables further reduces the differential in opioid prescribing rates across metropolitan and non-metropolitan areas to be less than half of a percentage point and statistically insignificant. These findings suggest that economic conditions, such as the type of working conditions that patients might experience, can interact with the healthcare system in unforeseen ways. For example, the combination of non-metropolitan areas having an industrial mix that skews towards jobs with higher injury rates plus a lack of access to alternative (non-opioid) pain treatments could account for a greater share of the disparity in opioid prescribing between rural (non-metropolitan) and urban (metropolitan) areas than either of these factors independently. and possibly give rise to more targeted interventions can reduce the persistent gap in opioid prescribing among more and less densely populated areas, with possible downstream impacts on overdose and mortality.

### Limitations

We acknowledge that there are several limitations to our research. First, since many county-level variables are included in the model, such as economic and healthcare-related indicators, there is the possibility of multicollinearity among them. To check for this, S6 Table reports Variance Inflation Factors (VIFs) that test whether the estimated coefficients are stable and unaffected by high correlation between variables. While some variables in the model are moderately correlated with each other, the VIFs are below 5 (with the exception of per capita hospital beds = 5.09) indicating that multicollinearity is not a severe problem.

Second, we cannot discount that there may be other confounding influences that could be fueling the differences in opioid prescribing that we observe by geography. For example, we are less able to explain geographical differences in receiving an opioid as a passenger in a car accident, a largely random event that is plausibly exogenous to underlying conditions that might require an opioid, suggesting that there may be confounding influences (e.g., drug-seeking behavior) from prior diagnoses that are not accounted for in our model. In particular, access to medication-assisted treatment (MAT) facilities and alternative therapies for opioid-use disorder are more limited in non-metropolitan areas, such that opioid use disorder may go untreated [12,31]. During the time period that we study, many non-metropolitan counties in the U.S. did not have any physicians that had gone through the training to obtain the necessary federal waiver to prescribe opioids as part of a medication-assisted treatment (MAT) protocol [32]. Although we control for differential access to treatments related to opioid-use disorder across rural versus urban areas, such as buprenorphine-naloxone, we are unable to fully account for all potential opioid variations used for MATss.

### Policy implications

Although at first glance there might seem to be little role for policy in addressing higher opioid prescribing in less densely populated areas, our results suggest some key areas for consideration. First, although demographics play a large role on the demand side, particularly veteran status, health insurance type is also an important factor. This suggests that there may be important differences in the coverage of alternative pain treatments (e.g., physical therapy) that could be addressed through health insurance regulation under the Affordable Care Act.

Second, there are complex interactions between supply and demand factors that suggest opportunities for more targeted opioid prescribing policies that account for local context. For example, medical boards might consider making occupational health care a continuing education requirement for primary care physicians who practice in areas with a high share of workers in physically demanding jobs.

Finally, there appears to be differences in physician practice behavior across geographic areas suggesting that providers become conditioned to follow local guidelines and practices [11]. State and federal agencies could consider implementing more specific guidelines for opioid prescribing (e.g., regulate the dosage in milligrams for specific conditions). These guidelines could be incorporated as part of the Rural Communities Opioid Response Program (RCORP), which addresses barriers to treatment for substance use disorder, including opioid use disorder. Alternatively, greater patient education about whether an opioid prescription is needed and/or desirable can help patients better advocate for their own pain management while managing the risks associated with opioid use.

## Conclusion

Overall, our findings suggest that demand-side factors play a greater role than those on the supply-side in explaining higher opioid prescribing rates among less densely populated areas. Moreover, demand- and supply-side factors can also interact in ways that contribute to the gap in opioid prescribing by geography beyond that of any single independent factor. Taken together, these findings suggest that more locally tailored interventions can reduce the persistent gap in opioid prescribing across metropolitan and non-metropolitan areas within a given state. Regardless of the policy strategy that is adopted, this study demonstrates that there are clear, tangible drivers that states can address in the near-term to reduce the persistent gap in opioid prescribing among more and less densely populated areas, with possible downstream impacts on overdose and mortality.

## Supporting information

S1 Fig Conceptual Model of Covariates. (TIF)

S1 Table Estimating the Relationship between Density and Opioid Prescribing: Alternative Measures. (PDF)

S2 Table Estimating the Relationship between Density and Opioid Prescribing: Joint Pain. (PDF)

S3 Table Estimating the Relationship between Density and Opioid Prescribing: Car Accidents. (PDF)

S4 Table Estimating the Relationship between Density and Opioid Prescribing Detailed Regression Coefficients: Back Pain (2010–2014). (PDF)

S5 Table Oaxaca Decomposition of Patient and County Factors for Back Pain Patients, Separate Models for Metropolitan and Non-Metropolitan Areas (2010–2014). (PDF)

S6 Table Variation Inflation Factors (VIFs) for Patient and County Level Variables. (PDF)

S7 Table Multi-Level Logistic Estimation of the Relationship between Density and Opioid Prescribing: Back Pain (2010–2014). (PDF)
