# Supplementary figures and images for: Why are Massachusetts opioid prescribing rates higher in rural versus urban areas?
Source: PLoS One. 2026 May 27;21(5):e0349247. doi: 10.1371/journal.pone.0349247 (PMC13215496; doi:10.1371/journal.pone.0349247)

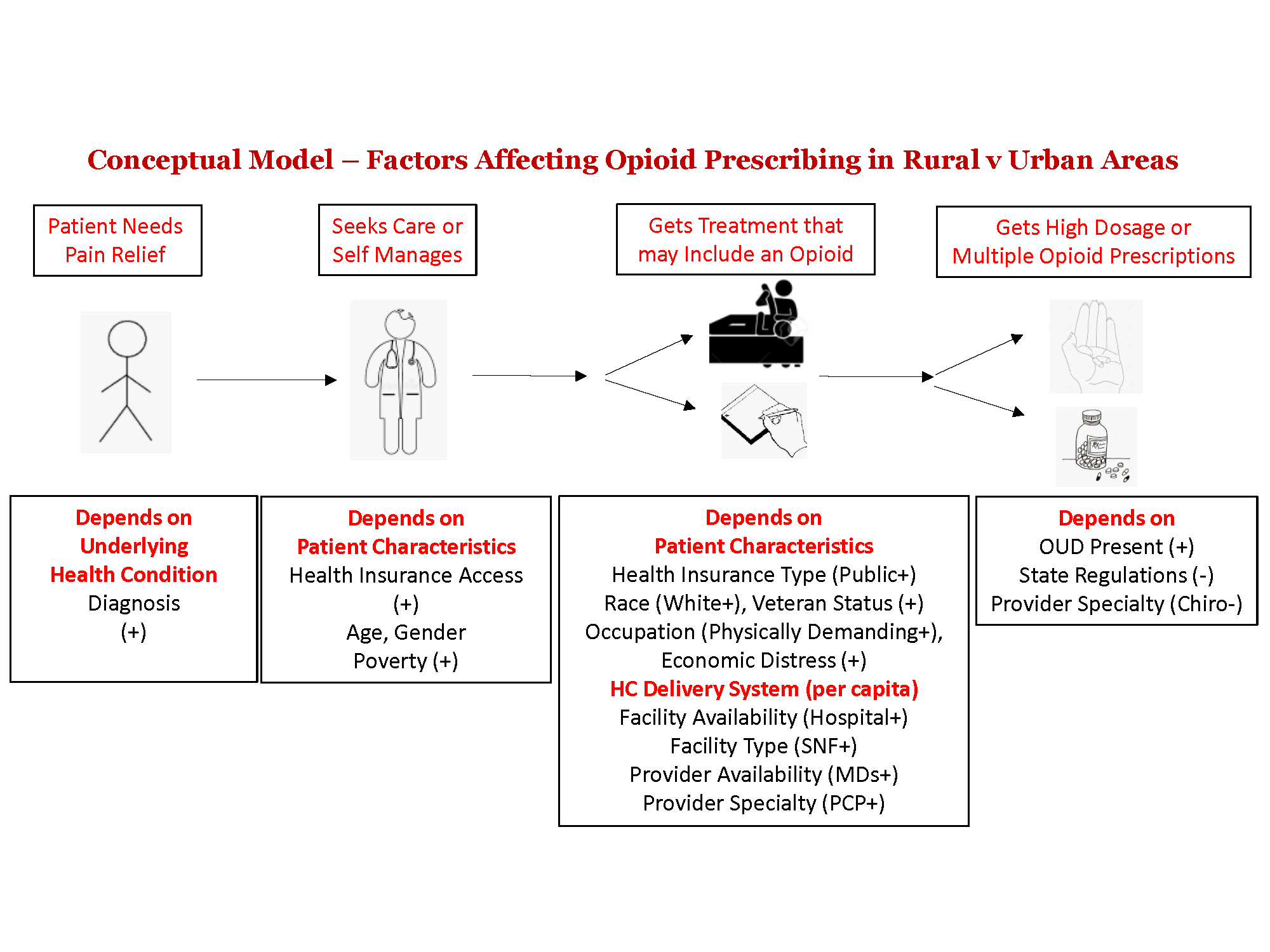

Supplement: S1 Fig — (TIF) [file pone.0349247.s001.tif]
