# Supplementary material for: Why are Massachusetts opioid prescribing rates higher in rural versus urban areas?
Source: PLoS One. 2026 May 27;21(5):e0349247. doi: 10.1371/journal.pone.0349247 (PMC13215496; doi:10.1371/journal.pone.0349247)
Supplement: S3 Table — (PDF) [file pone.0349247.s004.pdf]

Table S3. Estimating the Relationship between Density and Opioid Prescribing: Car Accidents

Panel A. Controlling for Patient Level Variables

	**Dependent Variable: Patient was Prescribed an Opioid (0/1)**				
	1	2	3	4	5
**Indicator for Non-Metropolitan (Rural) Area**	**0.041 *****	**0.038 *****	**0.033 *****	**0.039 *****	**0.024 *****
	**(0.001)**	**(0.001)**	**(0.001)**	**(0.001)**	**(0.001)**
Controlling for Patient Demographics	NO	YES	NO	NO	YES
Controlling for Patient Insurance Type	NO	NO	YES	NO	YES
Controlling for Patient Provider Specialty	NO	NO	NO	YES	YES
Number of observations	446,798	446,798	446,798	446,798	446,798
R-squared	0.002	0.074	0.087	0.046	0.119
Percent of urban-rural difference explained	-----	-6.1%	-19.8%	-4.3%	-40.1%

Panel B. Controlling for County Level Variables

	**Dependent Variable: Patient was Prescribed an Opioid (0/1)**				
	1	2	3	4	5
**Indicator for Non-Metropolitan (Rural) Area**	**0.041 *****	**0.031 *****	**0.036 *****	**0.019 *****	**0.018 *****
	**(0.001)**	**(0.001)**	**(0.001)**	**(0.001)**	**(0.001)**
Controlling for Population Demographics	NO	YES	NO	NO	YES
Controlling for Health Care Delivery System Variables	NO	NO	YES	NO	YES
Controlling for Economic Conditions	NO	NO	NO	YES	YES
Number of observations	446,798	446,798	446,798	446,798	446,798
R-squared	0.002	0.043	0.043	0.043	0.044
Percent of urban-rural difference explained	-----	-24%	-11%	-54%	-56%

Source: Patient-level variables are based on the authors' calculations using the Massachusetts All Payer's Claim Database and the Urban Area to ZIP Code Tabulation Area (ZCTA) Relationship File from the Census Bureau. County-level demographic and health care delivery system variables are from the Area Health Resource File. County-level labor market variables are from the American Community Survey.

Notes: See Table 1 for a list of covariates contained in each group. Each coefficient is from a separate regression.
***Indicates statistical significance at the one percent level, ** at the five percent level, and * at the ten percent level.
