# Supplementary material for: Why are Massachusetts opioid prescribing rates higher in rural versus urban areas?
Source: PLoS One. 2026 May 27;21(5):e0349247. doi: 10.1371/journal.pone.0349247 (PMC13215496; doi:10.1371/journal.pone.0349247)
Supplement: S5 Table — (PDF) [file pone.0349247.s006.pdf]

**Table S5. Oaxaca Decomposition of Patient and County Factors for Back Pain Patients, Separate Models for Metropolitan and Non-Metropolitan Areas (2010-2014)**

| | Dependent Variable: Patient was Prescribed an Opioid (0/1) | | | | | |
|---|---|---|---|---|---|---|
| | Metropolitan Areas | | | Non-Metropolitan Areas | | |
| | Coefficient | Standard Error | P> | Coefficient | Standard Error | P> |
| Independent Variable | | | | | | |
| Demographics | | | | | | |
| Percent male | -0.151 | 0.019 | 0.000 | -0.162 | 0.030 | 0.000 |
| Percent age 18-24 | 0.213 | 13.900 | 0.000 | 0.366 | 0.025 | 0.000 |
| Percent age 25-34 | 0.451 | 30.450 | 0.000 | 0.483 | 0.024 | 0.000 |
| Percent age 35-44 | 0.597 | 41.790 | 0.000 | 0.581 | 0.023 | 0.000 |
| Percent age 45-54 | 0.658 | 45.310 | 0.000 | 0.544 | 0.024 | 0.000 |
| Percent age 55-64 | -0.028 | -1.870 | 0.061 | -0.166 | 0.024 | 0.000 |
| Percent age 65+ | | (omitted) | | | (omitted) | |
| Male*Percent age 18-24 | 0.003 | 0.023 | 0.908 | -0.026 | 0.038 | 0.484 |
| Male*Percent age 25-34 | -0.006 | 0.022 | 0.803 | 0.053 | 0.036 | 0.145 |
| Male*Percent age 35-44 | 0.101 | 0.021 | 0.000 | 0.099 | 0.035 | 0.004 |
| Male*Percent age 45-54 | 0.143 | 0.022 | 0.000 | 0.175 | 0.035 | 0.000 |
| Male*Percent age 55-64 | 0.114 | 0.022 | 0.000 | 0.116 | 0.036 | 0.001 |
| Male*Percent age 65+ | | (omitted) | | | (omitted) | |
| Insurance Type | | | | | | |
| HMO/self-pay | 1.129 | 0.020 | 0.000 | 0.672 | 0.029 | 0.000 |
| PPO | 0.348 | 0.020 | 0.000 | -0.229 | 0.031 | 0.000 |
| Indemnity | -0.335 | 0.022 | 0.000 | -1.008 | 0.034 | 0.000 |
| Public (Medicare, Medicaid, VA) | 0.959 | 0.020 | 0.000 | 0.921 | 0.029 | 0.000 |
| Other (not specified) | | (omitted) | | | (omitted) | |
| Provider Speciality | | | | | | |
| Pain | -0.093 | 0.014 | 0.000 | 1.030 | 0.039 | 0.000 |
| Alternative pain treatment (e.g., chiropracter, physical therapy) | -0.217 | 0.120 | 0.000 | 0.048 | 0.021 | 0.021 |
| Addiction | 0.272 | 0.021 | 0.070 | 0.279 | 0.102 | 0.006 |
| Mental health | 0.360 | 0.016 | 0.000 | 0.364 | 0.033 | 0.000 |
| ER/critical care | 0.065 | 0.012 | 0.000 | 0.521 | 0.022 | 0.000 |
| Rehab | 0.000 | 0.014 | 0.000 | -0.038 | 0.022 | 0.081 |
| Dental | -0.023 | 0.023 | 0.973 | -0.067 | 0.023 | 0.003 |
| Surgery | -0.169 | 0.012 | 0.320 | 0.107 | 0.029 | 0.000 |
| General/family practitioner | -0.400 | 0.078 | 0.000 | 0.034 | 0.017 | 0.045 |
| Internal medicine | 0.063 | 0.010 | 0.000 | 0.192 | 0.173 | 0.266 |
| End of life (hospice, palliative care) | -0.254 | 0.040 | 0.000 | 0.056 | 0.016 | 0.000 |
| Diagnostic (e.g., radiology, pathology, immunology) | 0.360 | 0.010 | 0.000 | 0.085 | 0.050 | 0.089 |
| Non-MD (e.g., nurse, PA) | -0.519 | 0.365 | 0.000 | 0.356 | 0.015 | 0.000 |
| Medical Facility (e.g., hospital) | -0.048 | 0.010 | 0.155 | -1.589 | 0.465 | 0.001 |

| | | | | | | |
|---|---|---|---|---|---|---|
| Veteran Adminsitration / Military | -1.010 | 0.242 | 0.000 | -0.062 | 0.017 | 0.000 |
| Other | | (omitted) | | | (omitted) | |
| Population Demographics | | | | | | |
| Percent white | -1.010 | 0.242 | 0.000 | -1.089 | 0.390 | 0.005 |
| Percent persons under 65 without health insurance | 0.000 | 0.000 | 0.458 | 0.000 | 0.000 | 0.005 |
| Percent population veterans | 3.788 | 0.302 | 0.000 | 3.041 | 0.480 | 0.000 |
| Health Care Delivery System | | | | | | |
| Hospital beds per 10,000 residents | 12.629 | 2.544 | 0.000 | 83.933 | 9.647 | 0.000 |
| Skilled nursing facility beds per 10,000 population | 9.097 | 9.207 | 0.323 | -33.647 | 10.776 | 0.002 |
| Total active MDs per 10,000 population | 31.809 | 3.551 | 0.000 | -41.068 | 19.530 | 0.035 |
| General/family care specialists per 10,000 population | -393.744 | 67.502 | 0.000 | -62.180 | 122.657 | 0.612 |
| Economic Conditions | | | | | | |
| Unemployment rate, age 16+ | -1.062 | 0.800 | 0.184 | 0.244 | 1.173 | 0.835 |
| Poverty rate | -0.903 | 0.452 | 0.046 | -4.052 | 0.699 | 0.000 |
| Percent employed in production, transportation, material movir | 6.767 | 0.413 | 0.000 | 0.782 | 0.838 | 0.351 |
| Percent employed in natural resource, construction, maintenanc | 6.220 | 0.646 | 0.000 | -1.676 | 0.529 | 0.002 |
| Percent employed in service occupations | -3.969 | 0.649 | 0.000 | 1.965 | 0.883 | 0.026 |
| Year Dummies | | | | | | |
| 2010 | | (omitted) | | | (omitted) | |
| 2011 | 1.426 | 0.015 | 0.000 | 1.514 | 0.033 | 0.000 |
| 2012 | 1.436 | 0.019 | 0.000 | 1.583 | 0.037 | 0.000 |
| 2013 | 1.266 | 0.018 | 0.000 | 1.365 | 0.036 | 0.000 |
| 2014 | 1.315 | 0.023 | 0.000 | 1.237 | 0.035 | 0.000 |
| Number of observations | | 1,011,515 | | | 342,549 | |
| R-squared | | 0.089 | | | 0.100 | |

Source: Patient-level variables are based on the authors' calculations using the Massachusetts All Payer's Claim Database and the Urban Area to ZIP Code Tabulation Area (ZCTA) Relationship File from the Census Bureau. County-level demographic and health care delivery system variables are from the Area Health Resource File. County-level labor market variables are from the American Community Survey.

Notes: See Table 1 for a list of covariates contained in each group. Each coefficient is from a separate regression.
***Indicates statistical significance at the one percent level, ** at the five percent level, and * at the ten percent level.
