# Supplementary material for: Why are Massachusetts opioid prescribing rates higher in rural versus urban areas?
Source: PLoS One. 2026 May 27;21(5):e0349247. doi: 10.1371/journal.pone.0349247 (PMC13215496; doi:10.1371/journal.pone.0349247)
Supplement: S6 Table — (PDF) [file pone.0349247.s007.pdf]

Table S6. Variation Inflation Factors (VIFs) for Patient and County Level Variables

Panel A. Patient Level Variables	VIF
Lives in a non-metropolitan (rural) area	1.02
Percent male	4.97
Percent age 18-24	2.46
Percent age 25-34	2.73
Percent age 35-44	2.88
Percent age 45-54	3.05
Percent age 55-64	2.93
Male * Percent age 18-24	2.54
Male * Percent age 25-34	2.9
Male * Percent age 35-44	3.14
Male * Percent age 45-54	3.45
Male * Percent age 55-64	3.27
HMO/self-pay	1.51
PPO	3.9
Indemnity	4.35
Public (Medicare, Medicaid, VA)	3.91
Pain	1.08
Alternative pain treatment (e.g., chiropracter, physical therapy)	1.31
Addiction	1
Mental health	1.08
ER/critical care	1.19
Rehab	1.37
Dental	1.27
Surgery	1.09
General/family practitioner	1.47
Internal medicine	1.5
End of life (hospice, palliative care)	1.01
Diagnostic (e.g., radiology, pathology, immunology)	1.7
Non-MD (e.g., nurse, PA)	1.03
Medical Facility (e.g., hospital)	1.93
Veteran Adminsitration / Military	1

Panel B. County Level Variables	VIF
Percent white	3.96
Percent persons under 65 without health insurance	4.39
Percent population veterans	4.49
Hospital beds per 10,000 residents	5.09
Skilled nursing facility beds per 10,000 population	3.55
Total active MDs per 10,000 population	2.56
General/family care specialists per 10,000 population	1.98
Unemployment rate, age 16+	2.69
Poverty rate	4.46
Percent employed in production, transportation, material moving occupations	2.24
Percent employed in natural resource, construction, maintenance occupations	4.47
Percent employed in service occupations	4.67

Source: Patient-level variables are based on the authors’ calculations using the Massachusetts All Payer’s Claim Database and the Urban Area to ZIP Code Tabulation Area (ZCTA) Relationship File from the Census Bureau. County-level demographic and health care delivery system variables are from the Area Health Resource File. County-level labor market variables are from the American Community Survey.

Notes: VIF is the centered or uncentered variance inflation factors (VIFs) for the independent variables specified in a linear regression model that includes both patient- and county-level characteristics.
