# Supplementary material for: Why are Massachusetts opioid prescribing rates higher in rural versus urban areas?
Source: PLoS One. 2026 May 27;21(5):e0349247. doi: 10.1371/journal.pone.0349247 (PMC13215496; doi:10.1371/journal.pone.0349247)
Supplement: S7 Table — (PDF) [file pone.0349247.s008.pdf]

Table S7. Multi-Level Logistic Estimation of the Relationship between Density and Opioid Prescribing: Back Pain (2010-2014)

	Dependent Variable: Patient was Prescribed an Opioid (0/1)				
**Independent Variable**	Coefficient	Standard Error	P>	95% Confidence Interval	
Lives in a non-metropolitan (rural) area	0.006	0.001	0.000	0.005	0.007
Demographics					
Percent male	-0.005	0.002	0.002	-0.008	-0.002
Percent age 18-24	0.018	0.002	0.000	0.014	0.022
Percent age 25-34	0.059	0.002	0.000	0.056	0.062
Percent age 35-44	0.094	0.002	0.000	0.091	0.098
Percent age 45-54	0.119	0.002	0.000	0.116	0.122
Percent age 55-64	0.126	0.002	0.000	0.122	0.129
Percent age 65+		(omitted)			
Male*Percent age 18-24	-0.017	0.003	0.000	-0.023	-0.011
Male*Percent age 25-34	-0.021	0.003	0.000	-0.026	-0.017
Male*Percent age 35-44	-0.021	0.002	0.000	-0.025	-0.016
Male*Percent age 45-54	-0.005	0.002	0.022	-0.009	-0.001
Male*Percent age 55-64	0.004	0.002	0.062	0.000	0.009
Male*Percent age 65+		(omitted)			
Insurance Type					
HMO/self-pay	0.154	0.002	0.000	0.150	0.159
PPO	0.015	0.002	0.000	0.010	0.019
Indemnity	-0.059	0.003	0.000	-0.064	-0.054
Public (Medicare, Medicaid, VA)	0.152	0.002	0.000	0.147	0.156
Other (not specified)		(omitted)			
Provider Speciality					
Pain	0.233	0.003	0.000	0.227	0.239
Alternative pain treatment (e.g., chiropracter, physical therapy)	0.000	0.002	0.878	-0.003	0.004
Addiction	0.021	0.014	0.120	-0.006	0.048
Mental health	0.063	0.003	0.000	0.057	0.069
ER/critical care	0.085	0.002	0.000	0.080	0.089
Rehab	0.016	0.002	0.000	0.013	0.019
Dental	-0.035	0.011	0.001	-0.056	-0.014
Surgery	0.006	0.002	0.001	0.002	0.009
General/family practitioner	-0.009	0.001	0.000	-0.012	-0.006
Internal medicine	0.008	0.001	0.000	0.005	0.011
End of life (hospice, palliative care)	0.012	0.003	0.000	0.006	0.018
Diagnostic (e.g., radiology, pathology, immunology)	0.018	0.001	0.000	0.016	0.021
Non-MD (e.g., nurse, PA)	-0.013	0.005	0.012	-0.023	-0.003

Medical Facility (e.g., hospital)	0.073	0.001	0.000	0.070	0.075
Veteran Adminsitration / Military	-0.113	0.032	0.000	-0.176	-0.051
Other		(omitted)			
<u>Population Demographics</u>					
Percent white	-0.285	0.024	0.000	-0.332	-0.239
Percent persons under 65 without health insurance	-0.365	0.025	0.000	-0.415	-0.315
Percent population veterans	0.589	0.031	0.000	0.528	0.649
<u>Health Care Delivery System</u>					
Hospital beds per 10,000 residents	4.721	0.395	0.000	3.948	5.495
Skilled nursing facility beds per 10,000 population	-0.036	0.732	0.961	-1.471	1.400
Total active MDs per 10,000 population	5.755	0.508	0.000	4.759	6.751
General/family care specialists per 10,000 population	-3.780	6.528	0.563	-16.575	9.016
<u>Economic Conditions</u>					
Unemployment rate, age 16+	0.370	0.087	0.000	0.201	0.540
Poverty rate	-0.802	0.052	0.000	-0.903	-0.701
Percent employed in production, transportation, material moving occupations	1.485	0.064	0.000	1.360	1.610
Percent employed in natural resource, construction, maintenance occupations	0.700	0.058	0.000	0.585	0.814
Percent employed in service occupations	-0.470	0.065	0.000	-0.597	-0.343
<u>Year Dummies</u>					
2010		(omitted)			
2011	0.214	0.002	0.000	0.211	0.218
2012	0.222	0.002	0.000	0.217	0.226
2013	0.184	0.002	0.000	0.180	0.189
2014	0.197	0.003	0.000	0.192	0.202
Number of observations			1,354,064		

Source: Patient-level variables are based on the authors' calculations using the Massachusetts All Payer's Claim Database and the Urban Area to ZIP Code Tabulation Area (ZCTA) Relationship File from the Census Bureau. County-level demographic and health care delivery system variables are from the Area Health Resource File. County-level labor market variables are from the American Community Survey.

Notes: See Table 1 for a list of covariates contained in each group.
***Indicates statistical significance at the one percent level, ** at the five percent level, and * at the ten percent level.
